# Supplementary material for: Antigenic mapping and functional characterization of human New World hantavirus neutralizing antibodies
Source: eLife. 2023 Mar 27;12:e81743. doi: 10.7554/eLife.81743 (PMC10115451; doi:10.7554/eLife.81743)
Supplement: Figure 2—source data 1. [file elife-81743-fig2-data1.docx]

**Figure 2 - source data 1 (pertaining to Figure 2 panel c).** MAb binding in the presence of ANDV mutant constructs. The percent binding (% WT) of each mAb to the mutant constructs was compared to the WT SNV or ANDV control. The data are shown as average values from 3-4 independent experiments. All numberings for ANDV sequences was based on GenBank AF291703.2 and SNV sequences were based on GenBank KF537002.1.

| **Glycoprotein** | **ANDV-M variant** | **Monoclonal antibody** | | | | | | |
| --- | --- | --- | --- | --- | --- | --- | --- | --- |
|  |  | **ANDV-44** | **SNV-53** | **SNV-24** | **ANDV-5** | **ANDV-34** | **MIB22** | **JL16** |
| **Gn** | K67T | 74 | 51 | 54 | 84 | 106 | 0.4 | 103 |
|  | A96T | 105 | 58 | 100 | 110 | 109 | 107 | 110 |
|  | S97P | 48 | 96 | 85 | 100 | 103 | 100 | 106 |
|  | N108K | 75 | 49 | 55 | 109 | 109 | 97 | 111 |
|  | D121V | 57 | 39 | 54 | 104 | 110 | 101 | 110 |
|  | D121G | 79 | 66 | 79 | 115 | 111 | 112 | 113 |
|  | K124N | 92 | 75 | 71 | 114 | 108 | 111 | 107 |
|  | K225R | 80 | 56 | 50 | 96 | 110 | 101 | 106 |
|  | E231G | 57 | 32 | 30 | 37 | 92 | 25 | 74 |
|  | S309Y | 95 | 94 | 91 | 46 | 58 | 43 | 55 |
|  | D336Y | 86 | 58 | 66 | 78 | 15 | 15 | 104 |
|  | K356N | 25 | 53 | 46 | 105 | 117 | 110 | 114 |
| **Gc** | Y760F | 9 | -3 | 11 | 55 | 99 | 65 | 91 |
|  | D822E | 3 | -5 | -1 | 43 | 82 | 41 | 71 |
|  | K833N | 53 | 37 | 16 | 84 | 111 | 99 | 108 |
|  | P830S | 69 | 44 | 52 | 102 | 111 | 109 | 110 |
|  | S831Y | 79 | 76 | 39 | 100 | 105 | 98 | 108 |
|  | A871Q | 100 | 82 | 70 | 100 | 104 | 104 | 108 |
|  | S883R | 69 | 63 | 68 | 97 | 107 | 103 | 109 |
|  | N939D | 66 | 47 | 61 | 103 | 106 | 106 | 110 |
|  | N939H | 61 | 38 | 45 | 96 | 110 | 103 | 108 |
|  | A96T/Y760F | 110 | 31 | 115 | 98 | 110 | 109 | 108 |
|  | S97PK356N | 12 | 106 | 94 | 96 | 110 | 107 | 109 |
|  | E231G/A270D | 53 | 42 | 47 | -6 | 86 | 23 | 77 |
|  | S306Y/D336Y | 48 | 21 | 33 | 2 | 0.3 | 1 | 16 |
|  | D822E/K833N | 45 | 27 | -4 | 87 | 109 | 105 | 110 |
