# Supplementary material for: Antigenic mapping and functional characterization of human New World hantavirus neutralizing antibodies
Source: eLife. 2023 Mar 27;12:e81743. doi: 10.7554/eLife.81743 (PMC10115451; doi:10.7554/eLife.81743)
Supplement: Figure 2—source data 2. [file elife-81743-fig2-data2.docx]

**Figure 2 - source data 2 (pertaining to Figure 2 panel d)**

MAb binding in the presence of SNV mutant constructs. The percent binding (% WT) of each mAb to the mutant constructs was compared to the WT control. The data are shown as average values from 3-4 independent experiments. All numberings for SNV sequences were based on GenBank KF537002.1

| **Glycoprotein** | **SNV-M variant** | **Monoclonal antibody** | | |
| --- | --- | --- | --- | --- |
|  |  | **ANDV-44** | **SNV-53** | **SNV-24** |
| **Gn** | K86N | 100 | 81 | 102 |
|  | T312A | 93 | 101 | 86 |
|  | T312K | 99 | 102 | 117 |
|  | K357Q | 88 | 102 | 107 |
| **Gc** | P772L | 72 | 92 | 81 |
|  | K834N | 40 | 56 | 4 |
|  | K759E/P772L | 49 | 40 | 52 |
