# Supplementary material for: Antigenic mapping and functional characterization of human New World hantavirus neutralizing antibodies
Source: eLife. 2023 Mar 27;12:e81743. doi: 10.7554/eLife.81743 (PMC10115451; doi:10.7554/eLife.81743)
Supplement: Supplementary file 1. [file elife-81743-supp1.docx]

**Supplementary File 1**

|  |  | ANDV Gn^H^:Fabs ANDV-5:ANDV-34* | MAPV Gn^H^/Gc:Fabs SNV-24:SNV-53* | ANDV Gn^H^:Fabs ANDV-5/ANDV-34** |
| --- | --- | --- | --- | --- |
| Data Deposition | EMDB | EMD-26735 | EMD-26736 | EMD-27318 |
|  | PDB | -- | -- | 8DBZ |
| Microscope setting | Microscope | TF-20 | TF-20 | Glacios |
|  | Voltage (kV) | 200 | 200 | 200 |
|  | Detector | US-4000 | US-4000 | Falcon 4 |
|  | Mag | X50,000 | X50,000 | x190,000 |
|  | Pixel size | 2.18 | 2.18 | 0.73 |
|  | Exposure (e-/Å2) | 32 | 32 | 50 |
|  | Defocus range (μm) | 1.3-1.8 | 1.3-1.8 | 0.8-1.8 |
| Data | # Micrographs | 270 | 330 | 3100 |
|  | # particles |  |  | 306402 |
|  | # particle after 2D |  |  | 249853 |
|  | Final particles # | 44683 | 37800 | 208863 |
|  | Symmetry | C1 | C1 | C1 |
|  | Resolution FSC=0.143 | 17 | 22 | 4.1 |
| Model refinement and validation of Fabs** | Protein residues |  |  | 3643 |
|  | Map CC |  |  | 0.72 |
|  | RMSD |  |  |  |
|  | Bond lengths (A) |  |  | 0.004 |
|  | Bond angles |  |  | 0.677 |
|  | Ramachandran |  |  |  |
|  | Outliers (%) |  |  | 0 |
|  | Allowed (%) |  |  | 6.24 |
|  | Favored (%) |  |  | 93.76 |
|  | Poor rotamers (%) |  |  | 0 |
|  | MolProbity score |  |  | 2.38 |
|  | Clash score |  |  | 30.59 |
|  |  |  |  |  |
|  |  |  |  |  |
|  |  |  |  |  |
|  | *Negative stain data set | |  |  |
|  | **The ANDV Gn^H^ and the Fabs were docked to the map and rigid body refined | | | |

**Supplementary Table 1. Summary table of electron microscopy statistics.**
